# Gold Nanoparticle-Enhanced Detection of DNA Hybridization by a Block Copolymer-Templating Fiber-Optic Localized Surface Plasmon Resonance Biosensor

**DOI:** 10.3390/nano11030616

**Published:** 2021-03-01

**Authors:** Mengdi Lu, Wei Peng, Ming Lin, Fang Wang, Yang Zhang

**Affiliations:** College of Physics, Dalian University of Technology, Dalian 116024, China; mdlu@dlut.edu.cn (M.L.); linming9506@mail.dlut.edu.cn (M.L.); wangfang1020@mail.dlut.edu.cn (F.W.); yangzhang@dlut.edu.cn (Y.Z.)

**Keywords:** localized surface plasmon resonance, optical fiber, biosensor

## Abstract

To overcome low surface coverage and aggregation of particles, which usually restricts the sensitivity and resolution of conventional localized surface plasmon resonance (LSPR) fiber-optic sensors, we propose a simple self-assembled templating technique that uses a nanometer thickness block copolymer (BCP) layer of poly(styrene-b-4-vinylpyridine) to form a 33 nm gold nanoparticle (AuNP) monolayer with high uniformity and density for LSPR sensing. The LSPR resonance wavelength for this PS-b-P4VP templated methodology is 592 nm and its refractive index sensitivity is up to 386.36 nm/RIU, both of which are significantly improved compared to those of conventional LSPR techniques. Calibrated by a layer-by-layer polyelectrolyte deposition procedure, the decay length of this LSPR sensor is calculated to be 78 nm, which is lower than other traditional self-assembled LSPR sensors. Furthermore, hybridization between target ssDNA, which is linked with capture ssDNA on the LSPR biosensor and DNA–AuNP conjugates, leads to a low detection limit of 67 pM. These enhanced performances are significant and valuable for high-sensitivity and cost-effective LSPR biosensing applications.

## 1. Introduction

Localized surface plasmon resonance (LSPR) fiber-optic sensors have been widely investigated in recent years for biomolecule analysis [1,2], since the surface metallic nanoparticle arrays are extremely sensitive to the refractive index (RI) variation of the surrounding medium [3,4], which includes but is not restricted to their applications in antigen–antibody interactions [5,6] and biotin-streptavidin [7]. The material, shape, diameter and particle gap changes of the surface metallic nanoparticle arrays mainly affect the strength of the surrounding local electric field and greatly affect the sensitivity of LSPR applications [8,9]. The gap between particles of nanoparticle arrays, which is directly related to particle surface coverage and aggregation, is positively demonstrated by the adhesion layer between the self-assembled particle arrays and the substrate [10,11]. Currently, the self-assembly of metallic nanoparticle arrays for most LSPR fiber-optic sensors uses silane as a linking agent [12,13]. This method requires precise controlling of the self-assembly time and temperature, even though low-coverage and vast particle aggregations are still inevitable on particle arrays [14,15]. The complex manipulation process and lengthy workflow time are the major issues that limit fiber-optic LSPR sensor applications. Some polyelectrolyte alternate deposition methods are proposed to simplify the operating procedures for particle self-assembly [16,17]. This multilayer structure is used as the adhesion layer for nanoparticle fabrication while the deposition time is significantly reduced owing to the rapid electrostatic adsorption compared with the silane coupler method [14]. The surface coverage of particles is expected to be improved and the sensitivity of the polyelectrolyte-based LSPR sensor is increased accordingly; however, particle aggregations still exist when the surface coverage approaches a high level [18]. Specifically, the aggregations decrease the LSPR intensity, broaden its band, and strongly affect its RI sensitivity, resolution, and reproducibility [11]. Hence, a high-capacity self-assembly method is urgently required to fabricate distributed and uniform nanoparticle arrays for LSPR sensing [19].

The polymer templates have been recently reported to lead to well-dispersed and dense gold nanoparticle (AuNP) films with few aggregations on a flat surface. This method also works on curved surfaces; we explore it to fabricate LSPR substrates on optical fibers. Amphiphilic block copolymers at high concentrations (1–10 mg/mL) usually self-assemble in selective solvents to produce micelles with a wide range of morphologies on surfaces [20,21]. These block copolymer (BCP) micelles produce uniform nanoscopic arrays for the fabrication of high-density inorganic nanoparticles [22,23], such as AuNPs. BCP templates can be simply prepared by immersing the substrate in a dilute BCP solution, where the procedures and time of LSPR sensor fabrication are greatly reduced. Poly(styrene-b-4-vinylpyridine) (PS-b-P4VP), probably the most popular polymer template, is a suitable amphiphilic linear BCP to prepare template for the deposition of citrate capped AuNPs [24,25], owing to the electrostatic interactions between citrate and pyridine groups on P4VP blocks after protonation [26]. 

Metallic nanoparticles, such as AuNPs, are widely used as signal amplification tags for ssDNA hybridization owing to the light-scattering properties and the extremely large enhancement ability of the local electromagnetic field [27]. The particle-enhanced ssDNA hybridization is generally reported through use of surface plasmon resonance (SPR) with a continuous gold layer, and the signal amplification is mainly dependent on the volume effects of particles. However, AuNP-enhanced ssDNA hybridization based on LSPR sensors has rarely been reported. The electrical coupling between the AuNPs for signal amplification and the AuNPs on the substrate for LSPR sensing is stronger than the simple volume change for SPR.

Hence, we demonstrate a highly sensitive BCP-templated optical fiber LSPR biosensor for label-free ssDNA hybridization analysis. Compared with the traditional particle self-assembly methods (such as silane coupler and polyelectrolytes) and P4VP block-templating technique, this BCP-templating technique offers better sensitivity and decay length by forming uniform high-covered particle films with few aggregations. We apply it for high-sensitive ssDNA hybridization of the *rop* B gene. Rifampicin resistant tuberculosis, which relates to the *rop* B gene, is expected to be quickly identified by this BCP-templating LSPR fiber-optic biosensor.

## 2. Materials and Methods

### 2.1. Materials

Sodium citrate trihydrate, hydrogen tetrachloroaurate trihydrate (HAuCl_4_·3H_2_O), N-hydroxysuccinimide (NHS), 1-ethyl-3(3-dimethylaminopropyl) carbodiimidehydrochloride (EDC), 3-aminopropyl trimethoxy silane (APTMS) and chloroform tetrahydrofuran (THF) were obtained from (Aladdin Inc., Shanghai, China). Phosphate-buffered solution (PBS, pH = 7.4) and ssDNA strands were purchased from Sangon Biotech. 11-Mercaptoundecanoic acid (11-MUA), poly (4-styrene sulfonate) (PSS, 18 wt% in water, 75 kDa), and poly (allylamine) hydrochloride (PAH, 20 wt% in water, 65 kDa) were purchased from. (Sigma Aldrich Inc., St. Louis., MO, USA). Poly(4-vinyl pyridine) (P4VP, M_n_ × 10^3^: 36.3, M_w_/M_n_: 1.17) and poly(styrene)-*b*-poly(4-vinyl pyridine) (PS-*b*-P4VP, M_n_×10^3^: 41-b-20, M_w_/M_n_: 1.18) were obtained from Polymer source. Plastic cladding silica multimode fiber (HP 400/430-37/730E) was purchased from (YOFC Inc., Wuhan, China). Milli-Q water was used throughout the experiments.

### 2.2. Preparation of the Fiber-Optic LSPR Sensor

**Figure 1:**
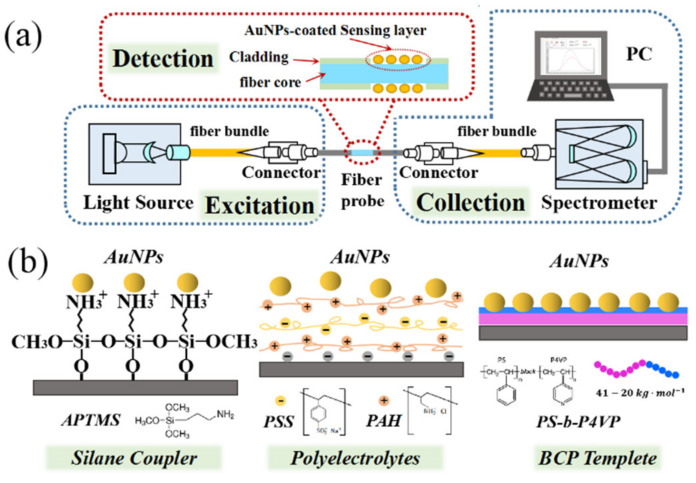
Schematic diagram of (**a**) gold nanoparticle (AuNP)-based fiber-optic localized surface plasmon resonance (LSPR) sensor; (**b**) AuNP self-assembly principle bases on silane coupler, polyelectrolytes and block copolymer (BCP)-templating technique, respectively.

The preparation process of fiber-optic sensor is shown in Figure 1. A 10 cm length fiber with 8 mm cladding removed in the middle was used as the sensing element. The unclad bare fiber was then immersed in Piranha solution (the volume ratio of H_2_SO_4_:H_2_O_2_ is 3:1—this is an extremely dangerous solution) for 15 min to clean and hydroxylate the fiber surface and was then rinsed with Milli-Q water.

The AuNPs were synthesized via the seeded growth method [28]. After synthesis, the gold colloid solution was centrifuged and redistributed in Milli-Q water (pH = 5.0). The cleaned fibers were first fabricated with particle junction layers. The junction layers were obtained from 10% of APTMS methanol solution (immersed for 2 h at 40 ℃), polyelectrolyte solutions (alternately deposited in 1 mg/mL of PAH and 1mg/mL of PSS for 15 min each), and 0.05 mg/mL PS-b-P4VP (or P4VP) THF solution (dip-coating 10 min), respectively. The fibers were then rinsed by Milli-Q water and dried. After this, the fibers were immersed in gold colloid solution for 2 h, then rinsed with Milli-Q water again and treated by oxygen plasma etching with a power of 9 W for 30 s to remove the connection layer from nonparticle fixation areas.

### 2.3. Decay Length Detection

An HL-2000 light source (Ocean Optics) with a wavelength range of 360–2000 nm was used to transmit light and an HR4000 Series Spectrometer (Ocean Optics) was used to receive the spectrum signal.

A layer-by-layer polyelectrolyte deposition method was employed to achieve the decay length of the proposed LSPR fiber sensor. A PAH solution (cationic polyelectrolyte, 1 mg/mL with 0.1 M NaCl, pH 5.0) and a PSS solution (anionic polyelectrolyte, 1 mg/mL with 0.1 M NaCl, pH 4.0) were fabricated on the sensing surface for 1 min in turn to obtain one PAH/PSS bilayer. A water washing step was performed after each layer deposition. The positively charged PAH layer was directly formed on the negatively charged sensing surface of AuNPs. 

### 2.4. Bio-Application of AuNP-Enhanced DNA Hybridization

A density 11-MUA monolayer self-formed on the AuNP-based LSPR fiber sensor through a 12 h immersion in the mercaptan solution (10 mM 11-MUA in ethanol). Then, 3 μΜ capture ssDNA (3′-CAA CCC GGG GA/5AmMC6/-5′), which is a half-complementation of target *rop* B ssDNA (3′-TCC CCG GGT TGT AGC CAG ACT A-5′), was coupled to the 11-MUA linkers via dehydration condensation reaction between amine and carboxylate groups with EDC/NHS (0.1 M/0.1 M) activation for 30 min. 

A thiol-labeled probe ssDNA (3′-/3ThioMC3-D/AAA AAA TAG TCT GGC TA-5′), which is a complementation to the other half-sequence of *rop* B ssDNA, was selected to prepare DNA/AuNP conjugates for biosensing signal amplification (the detail of preparation method has been reported in previous work [29]) and the diameters of AuNPs are 15 nm. Different concentrations of *rop* B ssDNA were hybridized with capture ssDNA for 20 min and washed with PBS for 10 min. The DNA/AuNP conjugates were used to enhance the LSPR signal for 1 h. For the control experiment, we used double mismatch *rop* B ssDNA (3′-TCC CCG G**C**T TGT AGC C**T**G ACT A-5′) and noncomplementary *kat* G ssDNA (3’-GTG TTC AGC CCA CCC TCC AGT T-5’) for comparison. 

## 3. Results

### 3.1. Surface Characterization of the LSPR Sensor

**Figure 2:**
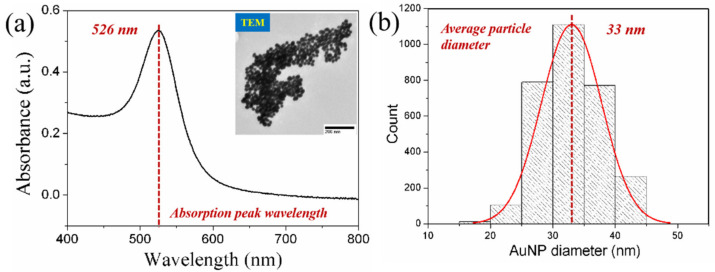
Gold nanoparticle characterization. (**a**) UV–vis spectrum and (**b**) diameter distribution of AuNPs of average diameters.

To evaluate the surface morphology of the BCP-templated AuNP film on the fiber, two other standard methods based on silane coupler and polyelectrolytes were performed for the self-assembly film of 33 nm AuNPs. The diameters of AuNPs were determined with a UV–Vis spectrum and the transmission electron microscopy (TEM) image can be seen in Figure 2. The diameters and concentration of particles could be calculated by the UV–vis spectrum according to the method reported by Haiss et al. [30]. We used the ratio of the absorbance of AuNPs at the surface plasma resonance peak (A_spr_) to the absorbance at 450 nm (A_450_) to calculate the particle diameters. The particle concentration (mol per liter) could also be estimated through A_450_/ε_450_, while ε_450_ is molar decadic extinction coefficient at 450 nm. As we show in Figure 2a, the diameters and concentration of AuNPs are calculated as 33 nm and 0.12 nm. AuNPs with uniform sizes were observed from the TEM image and the average particle diameter distribution is shown in Figure 2b, without the obvious presence of small or large impurity nanoparticles, which are functionalized on the fiber for LSPR sensing.

**Figure 3:**
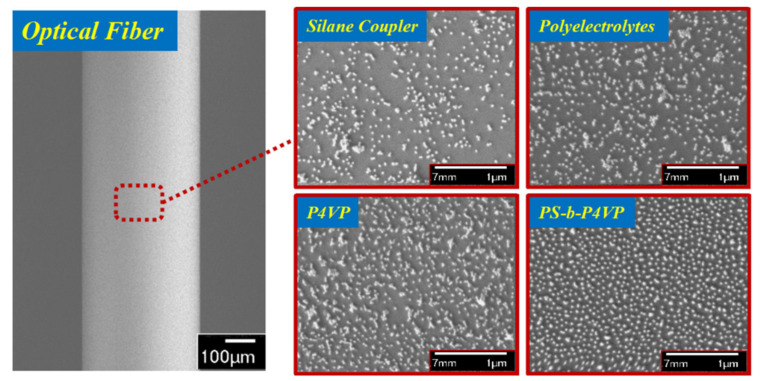
The scanning electron microscopy (SEM) images of AuNP-coated fiber surfaces which were deposited by APTMS, polyelectrolytes, P4VP and the PS-b-P4VP monolayer, respectively.

The surface morphologies of the AuNP films were assessed by scanning electron microscopy (SEM). Figure 3 shows the SEM images of the AuNP-coated fiber surfaces using silane coupler, polyelectrolytes, P4VP and PS-b-P4VP brush-like monolayer, respectively. The particle distribution of these kinds of AuNP films are uniform over a large area, but the mass of particle aggregations was still widely observed for the silane coupler and polyelectrolytes methods. The P4VP block without a PS block could not disperse the particles from each other, leading to a large number of aggregations. For the PS-b-P4VP, the P4VP chains worked as particle linkers while the PS chains protected particles from agglomeration since they only floated around on the surface without binding. The SEM results indicate that the particle distribution of our BCP-templated fiber is densely arranged and well-dispersed. Moreover, the surface coverage of particles is also an important factor affecting the performances of the LSPR sensor. The BCP-templated surface has the maximum coverage fraction of AuNPs (18.89% ± 0.36), compared to the P4VP (16.91% ± 0.48), silane coupler (13.31% ± 0.53) and polyelectrolyte-based (16.38% ± 0.42) fiber surfaces. The higher surface coverage of particles means more particles are immobilized on the surface and there is a larger surface area to bind biomolecules—the LSPR signal would increase accordingly.

### 3.2. Refractive Index Sensitivity

According to the surface morphology results, it is obvious that the BCP chains of PS-b-P4VP play an ideal role in the self-assembly of particles to significantly improve the surface coverage and drastically eliminate the aggregation of particles. The gold colloid solution for deposition was adjusted to acidity (pH 5.0), and the amino groups on P4VP blocks were protonated with a maximum surface charge to combine negative charges AuNPs via electrostatic interactions, so that the coverage of AuNPs approaches a maximum level [31]. When the surface coverage reaches the maximum value, the attractions between particles and fiber surface and the repulsive forces of adjacent particles reach equilibrium [14]. The hydrophobic PS blocks which are not linked to AuNPs or the fiber surface could prevent the aggregation of particles.

**Figure 4:**
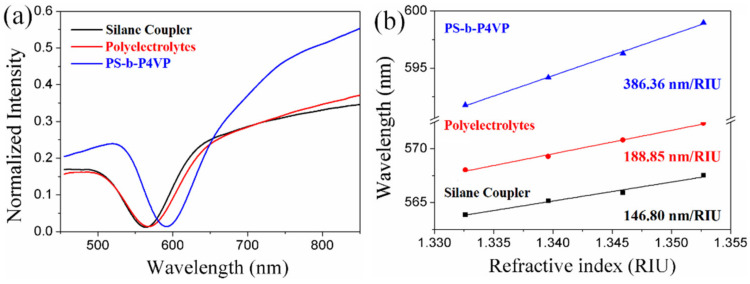
Comparison of (**a**) the LSPR spectra and (**b**) refractive index sensitivity of the sensors which were prepared by silane coupler, polyelectrolytes and BCP monolayers.

Figure 4 compares the LSPR spectra and RI sensitivity for these three methods (silane coupler, polyelectrolytes, and BCP-templating technique). The LSPR spectral characteristics presented in Figure 4a are in high agreement with the surface morphologies in Figure 3; the dense AuNP film with few aggregations of the BCP-templated LSPR sensor led to a dramatic decrease in the full width to half of the maximum and an increase in the resonance depth. As we predicted, the RI sensitivity for the BCP-templated (386.36 nm/RIU) LSPR sensor was found to increase by more than 2-fold than those made by the silane coupler (146.80 nm/RIU) and polyelectrolytes (188.85 nm/RIU). These results illustrate that the low surface coverage and high aggregations of particles will decrease the RI sensitivity and lead to poor sensing performances. Our BCP-templating technique simplifies the manufacturing and preparation procedures, quickly prepares a high-capacity AuNP film and highly increases the sensitivity of the LSPR sensor. Furthermore, this dense high surface coverage particle self-assembly method, with no aggregations, could potentially be applied as an effective platform for preparation of micro–nano structures over a large area.

### 3.3. Surface Sensitivity

**Figure 5:**
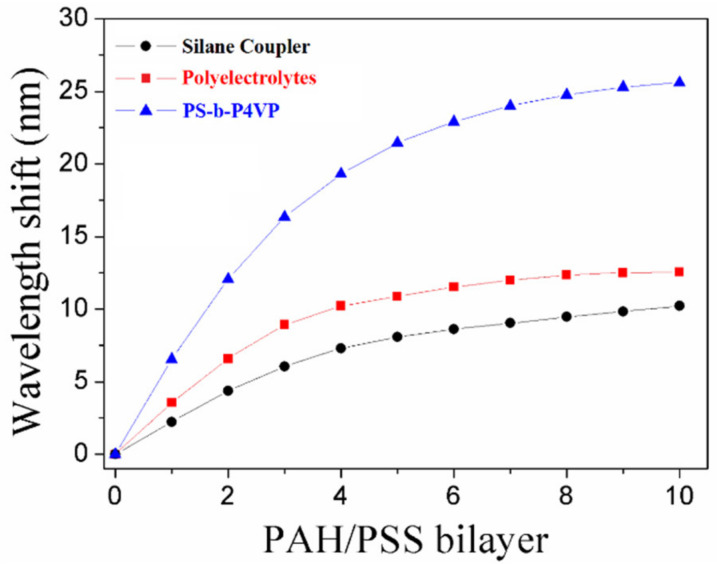
Plasmonic wavelength shift of LSPR peaks from successive adsorption of 10 poly (allylamine) hydrochloride (PAH)/poly (4-styrene sulfonate) (PSS) bilayers to assess the decay length for different deposition methods.

We measured surface sensitivity with the adsorption of PAH/PSS bilayers. A relatively monotonic increase in the plasmonic wavelength shift was observed for each PAH/PSS bilayer with a low number of bilayers, which started to plateau when a higher number of bilayers was reached (Figure 5). The decay length (l*_d_*), which depends on the type of metal and physical parameters of the nanostructure, could be estimated by

(1) $$\Delta \lambda =S\Delta n\left[1-exp\left(\frac{-2d}{l_{d}}\right)\right]$$

where Δλ is the LSPR wavelength shift (nm), S is the RI sensitivity (nm/RIU) and Δn is the RI difference between the liquid medium (n_water_ = 1.33) and the adsorbate layer (n_layer_) of thickness d. The RI of PAH/PSS bilayer could be estimated to 1.46 and the thickness of per bilayer is 2.9 ± 0.8 nm [29]. Additionally, we assumed that S and l_d_ remained constant during the growth of the bilayer in our case. The decay lengths associated with different deposition methods were calculated by Equation (1). A shorter decay length is ideal for molecular adsorption events which lead to more sensitive surface electric field changes. The average decay lengths were estimated to be 78, 84 and 86 nm, respectively, for PS-b-P4VP, polyelectrolytes and APTMS monolayers. Hence, BCP-templated LSPR fiber sensors with short decay length are beneficial for high-surface sensitivity detection of biomolecules.

### 3.4. Biosensing Application for ssDNA Hybridization

**Figure 6:**
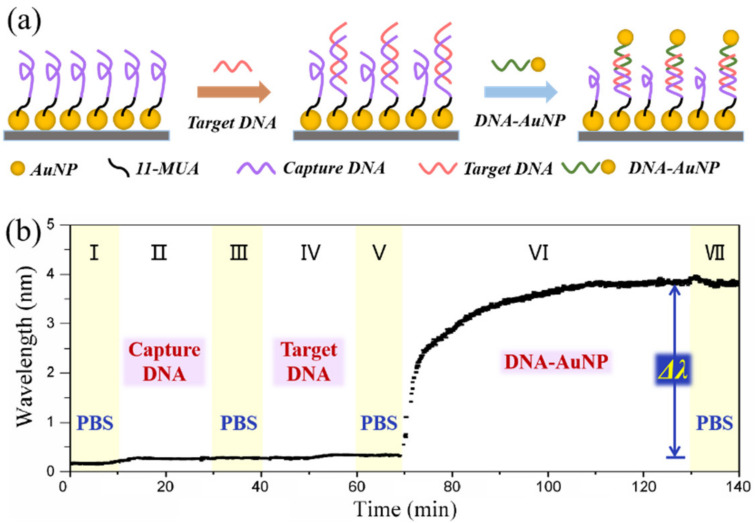
AuNP-enhanced LSPR biosensing of ssDNA hybridization of the LSPR biosensor. (**a**) Schematic diagram of hybridization process. (**b**) Wavelength responses and calibration binding curve for ssDNA hybridization.

In order to demonstrate the potential biosensitivity of our proposed BCP-templated LSPR fiber sensor, a sandwich configuration was established to achieve the direct detection assay for *rop* B gene analysis. The experimental process is described in Figure 6a; the capture ssDNA consisting of half of the complementary sequence of *rop* B ssDNA was first bound to the AuNP film with 11-MUA linker and *rop* B ssDNA was bound on the capture ssDNA. Then, the other half of the complementary DNA/AuNP conjugates were employed to enhance the LSPR signal. As shown in Figure 6b, we obtained almost no wavelength shift of capture ssDNA and target ssDNA binding processes, but there was a dramatically large wavelength shift with DNA/AuNP conjugates to amplify the sensing signal.

**Figure 7:**
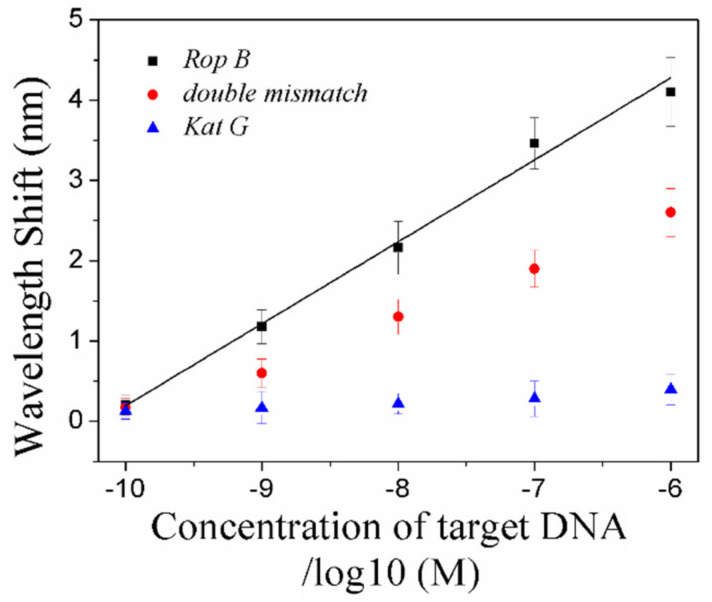
The LSPR wavelength shifts and linear calibration curve after AuNP/DNA amplification for various concentrations of rop B ssDNA. The wavelength shifts of double mismatch rop B ssDNA and kat G ssDNA are also shown for negative experiments.

In Figure 7, the plasmonic shift of DNA/AuNP solution grew linearly with the increasing concentration of complementary *rop* B ssDNA with a coefficient of determination (R^2^) of 0.985, the concentration of *rop* B was achieved in a wide range of 10^−10^–10^−6^ M and the limit of detection decreased to 67 pM. Negative control experiments with double mismatch *rop* B and noncomplementary *kat* G were carried out in the same conditions. The response from double mismatch *rop* B is much smaller than that of *rop* B, but much higher than that of *kat* G. These results demonstrate that the positive wavelength shift of *rop* B is effective and unique and that the nonspecific adsorption of DNA/AuNPs is negligible. 

## 4. Conclusions

In conclusion, we designed and demonstrated a simple self-assembled AuNP-coated LSPR optical fiber biosensor that exploited BCP brush-like monolayers to monitor label-free ssDNA hybridization. This BCP-templated AuNP monolayer presented uniform and high surface coverage of particles without aggregation, leading to high sensitivity and resolution for LSPR sensing, which has unique performance advantages compared to other deposition methods including those using silane couplers, polyelectrolytes and P4VP. This BCP-templated LSPR fiber-optic biosensor provides high RI sensitivity (386.36 nm/RIU) and a low decay length (78 nm), which indicates its high surface sensitivity for molecular adsorption. Thereby, we employed this sensor for label-free *rop* B ssDNA hybridization by using DNA/AuNPs as signal amplification tags and achieved an extensive dynamic range from 10^−10^ to10^−6^ M and a low limit of detection of 67 pM. This BCP-templating technique possesses enhanced LSPR characteristics and has great potential and values for highly sensitive biomolecule detection in practical applications.

## Data Availability

Data are available in the main text.
